# Improving long short-term memory (LSTM) networks for arbitrage spread forecasting: integrating cuckoo and zebra algorithms in chaotic mapping space for enhanced accuracy

**DOI:** 10.7717/peerj-cs.2552

**Published:** 2024-12-12

**Authors:** Mingfu Zhu, Yaxing Liu, Panke Qin, Yongjie Ding, Zhongqi Cai, Zhenlun Gao, Bo Ye, Haoran Qi, Shenjie Cheng, Zeliang Zeng

**Affiliations:** 1 Henan Polytechnic University, School of Computer Science and Technology, Jiaozuo, Henan, China; 2 Hebi National Optoelectronic Technology Co, Ltd., Hebi, Henan, China

**Keywords:** LSTM networks, Arbitrage spread forecasting, Hyperparameter setting, Cuckoo algorithm, Zebra algorithm

## Abstract

Long short-term memory (LSTM) networks, widely used for financial time series forecasting, face challenges in arbitrage spread prediction, especially in hyperparameter tuning for large datasets. These issues affect model complexity and adaptability to market dynamics. Existing heuristic algorithms for LSTM often struggle to capture the complex dynamics of futures spread data, limiting prediction accuracy. We propose an integrated Cuckoo and Zebra Algorithms-optimised LSTM (ICS-LSTM) network for arbitrage spread prediction. This method replaces the Lévy flight in the Cuckoo algorithm with the Zebra algorithm search, improving convergence speed and solution optimization. Experimental results showed a mean absolute percentage error (MAPE) of 0.011, mean square error (MSE) of 3.326, mean absolute error (MAE) of 1.267, and coefficient of determination (R2) of 0.996. The proposed model improved performance by reducing MAPE by 8.3–50.0%, MSE by 10.2–77.8%, and MAE by 9.3–63.0% compared to existing methods. These improvements translate to more accurate spread predictions, enhancing arbitrage opportunities and trading strategy profitability.

## Introduction

In today’s data-intensive world, the ability to process vast amounts of real-time data efficiently is paramount (Engelmayer, Georgiev & Veličković, 2024). Against this backdrop, the latest data from the China Futures Association shows that in 2023, the scale of China’s futures market has steadily expanded. The cumulative trading volume in the futures market reached 8.501 billion lots, with a cumulative turnover of 56.851 trillion dollars, representing year-on-year growth of 25.60% and 6.28% respectively (Liu, Feng & Xu, 2024). This robust expansion not only showcases the vitality of China’s futures market but also underscores the pressing need for advanced data processing techniques to handle the ever-growing volumes of real-time market data. Artificial intelligence (AI) has ushered in a revolutionary era for the financial industry, propelling unprecedented advancements in data analysis and parallel computing. This technological leap has inspired numerous researchers to delve deeper into time series forecasting (TSF) problems (He et al., 2023). It also paved the way for the emergence and development of various trading strategies, including statistical arbitrage. Statistical arbitrage, a common type of algorithmic trading, is widely recognized and utilized in academic and industrial environments. Given the potential to generate higher profits with limited risk, an increasing number of investors are transitioning into arbitrageurs and entering the arbitrage market. However, intense competition has led to a decline in the profitability of statistical arbitrage. Kozhan & Tham (2012) pointed out that the proliferation of competitive arbitrageurs results in higher execution risk, as highlighted in their research. Consequently, enhancing the efficiency of trading strategies has become a crucial factor in determining arbitrage profitability.

However, most futures arbitrage indices behave very similarly to a random walk because the financial time series data is noisy and non-stationary (He et al., 2023). Undoubtedly, it is very difficult to predict the futures arbitrage market, since the volatility is too large to be captured in a model (Olorunnimbe & Viktor, 2023). Despite these difficulties, there has been a constant desire to develop a reliable futures arbitrage market prediction model (Mahmoodi et al., 2023). Several approaches have been made in recent decades to forecast future arbitrage markets using statistics and soft computing techniques. Earlier studies relied on statistical methods, but they often struggled with complex financial data due to computational complexities and efficiency constraints (Vullam et al., 2023). Accordingly, various machine learning techniques, such as artificial neural networks (ANN) and support vector machines (SVM), which can capture nonlinearity and complex characteristics of financial time series, have started to be utilized for financial market prediction. These approaches have improved the ability to predict the chaotic environments of future arbitrage markets by capturing their nonlinear and unstructured nature (Basterrech & Rubino, 2023; Adebiyi, Adewumi & Ayo, 2014). Unlike traditional statistical models, machine learning models are data-driven, non-parametric weak models, and they let “the data speak for themselves.” Consequently, machine learning models are less susceptible to the problem of model misspecification compared to most parametric models.

In recent years, there have been increasing attempts to apply deep learning techniques to financial market prediction. The Local Linear Embedding Dimensionality Reduction (LLE) algorithm (Yu et al., 2020) was chosen to reduce the dimensionality of the factors affecting the stock price and the dimensionality reduced data is used as a new input variable to the back-propagation (BP) neural network for stock price prediction. Khoobjou & Mazinan (2017) proposed a financial prediction model based on the generalized regression neural network method. This method can improve the operation speed, and the prediction performance is also better than that of the traditional BP neural network. Jahan & Sajal (2018) used a recurrent neural network (RNN) algorithm on the time-series data of a stock and also cross-checked the predicted closing price with the true closing price. This demonstrates that RNNs can be used to predict other volatile financial instruments (Jahan & Sajal, 2018). The temporal representation capabilities of RNNs offer advantages in tasks that involve processing sequential data, such as financial predictions, natural language processing, and speech recognition (Weerakody et al., 2021). In contrast to recurrent neural networks (RNNs), conventional neural networks exhibit limitations in handling sequential data due to the absence of memory mechanisms essential for capturing temporal dependencies and dynamics within the data. Wang et al. (2018) studied the application of long short-term memory (LSTM) networks in the stock market. They defined a parameter combination library and used the skill of dropout to get the more ideal prediction results (Wang et al., 2018).

Over the past 2 years, Huang et al. (2024) introduced a novel deep network architecture called bidirectional long short-term memory (BiLSTM)-Attention, which enhances the network’s ability to identify key features and patterns in stock market data, allowing the model to focus on the most relevant aspects of the data. Yang et al. (2023) proposed an end-to-end model called DRL-UTrans for learning a single stock trading strategy that combines deep reinforcement learning, transformer layer, and U-Net architecture, which provides higher profitability compared to seven baseline methods. Yañez, Kristjanpoller & Minutolo (2024) investigate the performance of a Transformer-based deep neural network in predicting financial market returns, compared to a conventional model. The study employs a comprehensive approach, including iterative abandonment testing and batch size optimization, to improve the model’s predictive performance. The results show the proposed Transformer variant outperforms the benchmark model in all cases (Yañez, Kristjanpoller & Minutolo, 2024). Zhu et al. (2024) proposed a hybrid model PMANet for stock price prediction. PMANet incorporates multiscale temporal feature convolution and ant particle swarm optimization, improving understanding of stock data dependencies. Experimental results show PMANet’s prediction closely matches actual values, with feasible and generalized predictive capability (Zhu et al., 2024). Xue, Li & Wang (2024) investigates a novel composite prediction framework that integrates the variational mode decomposition (VMD), PatchTST, and adaptive scale weighting layer (ASWL). The VMD-PatchTST-ASWL framework significantly improves the prediction accuracy and exhibits robust performance in different indices compared to conventional models (Xue, Li & Wang, 2024). Shi (2024) proposes MambaStock, a Mamba-based stock price prediction model with a selection mechanism and scanning module. MambaStock efficiently mines historical data to predict future stock prices, outperforming previous methods with highly accurate forecasts (Shi, 2024).

While the above studies have made significant progress in time series forecasting, LSTM performs particularly well in the specific application scenario of futures arbitrage. Zaheer et al. (2023) propose a hybrid deep learning prediction model. The model takes input stock data and predicts two stock parameters for the next day: closing price and high price. The results show that LSTM outperforms convolutional neural network-long short-term memory (CNN-LSTM), CNN-RNN outperforms CNN-LSTM, CNN-RNN outperforms LSTM, and the proposed single-layer RNN model beats all other models (Zaheer et al., 2023). Lahboub & Benali (2024) proposed a data-driven approach to predict stock prices on the Moroccan stock exchange, testing three prediction models: the ARIMA, LSTM, and transformers, applied to historical stock price data of three well-known credit companies (EQD, LES, and SLF) listed on the Casablanca stock exchange, the results show that LSTM model achieves the highest level of accuracy. Han & Li (2024) propose a model to optimize arbitrage spreads based on Long Short-Term Memory (LSTM). The training results show that the proposed optimization model can successfully filter out unprofitable trades and significantly outperforms the returns of the benchmark strategy and the CSI 300 index in the same period (Han & Li, 2024). From the above study, we can conclude that LSTM is more suitable for more suitable for arbitrage spread prediction.

Although LSTM shows good performance in the arbitrage spread prediction problem, the biggest challenge in LSTM neural networks is tuning their hyperparameters, namely batch size, number of epochs, learning rate, and optimizing the connection weights as well as the bias of the network. The automatic exploration of neural network architectures has been attempted by several authors. Sakshi & Kumar (2019) utilized genetic algorithms to optimize the parameters of an artificial neural network (ANN) and discovered that the proposed model exhibits a brief training period, fast convergence, and a higher success rate. Peng et al. (2018) applied differential evolution (DE) to obtain the optimal values of various hyperparameters, such as window length, the number of hidden nodes, batch size, and the number of epochs in LSTM for electricity price prediction. Liu & Liu (2019), incorporated a modified genetic algorithm (GA) to select the optimal feature subset and hidden neurons of LSTM neural networks for house price prediction in China. Hao, Song & Du (2023) utilized a two-layer LSTM with time attention to encode stock data and focus on temporal dependencies. In addition, the proposed adaptive particle swarm optimization algorithm is used to select the key parameters of the network structure, which improves the overall prediction performance (Hao, Song & Du, 2023). Liu et al. (2023) used the Cuckoo Search algorithm (CS) to optimize an LSTM neural network to predict four indicators of the reservoir in 2022. The experimental results show that the average absolute error and root mean square error of the CS-LSTM-based prediction model is lower than that of the comparison model, and the coefficient of determination is higher than that of the comparison model and better than that of the LSTM model (Liu et al., 2023). Rajalakshmi & Kala (2024) used CS optimization to find the best weights and bias values for LSTM networks to provide appropriate decision-making to the driver, and the results propagated to show that CS improves the performance of LSTM. Rani & Kavitha (2024) used the Zebra Optimisation Algorithm (ZOA) to fine-tune the EEG signal features. The features obtained from the selection algorithm were then fed into a hyperparameter-optimized LSTM classifier to classify normal and abnormal seizures, The results show high accuracy for three datasets (Rani & Kavitha, 2024). Shahul Hameed & Ravi (2022) proposed an improved CS optimization model to tune the hyperparameters of the LSTM model. In comparative evaluation with related benchmark techniques, namely genetic algorithm optimized LSTM, particle swarm optimized LSTM, and CS optimized LSTM, the result shows that the recommended methodology outperforms the taken benchmark models and provides better accuracy (Shahul Hameed & Ravi, 2022). We can see in Table 1 a comparison of the main methods mentioned above.

**Table 1:** Summary of key literature.

Authors (Year)	Input data	Output	Method
Liu & Liu (2019)	Eight characteristics of the housing market in Shenzhen, China	House price	GA-LSTM
Hao, Song & Du (2023)	Stock data set	Stock price	APSO-TA-LSTM
Liu et al. (2023)	Four indicators for reservoirs	Water quality	CS-LSTM
Rani & Kavitha (2024)	Three standard datasets such as Temple University Hospital (TUH), Bonn University (BU) and Bern Barcelona (BB)	Classify the normality and abnormality of seizures	ZOA-LSTM
Shahul Hameed & Ravi (2022)	Technical indicators extracted from Bitcoin’s Historical Data	Bitcoin price	MCSO-LSTM

The CS algorithm has shown strong performance on a variety of optimization problems, but it still has limitations, especially in dealing with complex problems and large-scale data (Yang & Deb, 2009). One main drawback is its reliance on Lévy flights, which enhances global search but often results in insufficient local search, causing the algorithm to get stuck in local optima. Additionally, CS tends to have slower convergence in the early stages, particularly for high-dimensional problems, limiting its effectiveness in financial time series forecasting. To address these issues, this study proposes the Integrated Cuckoo Search (ICS) algorithm, which builds on the cuckoo algorithm but introduces key innovations. The ICS algorithm incorporates a “zebra” pattern search strategy to achieve a better balance between global exploration and local exploitation, overcoming the large jumps in Lévy flights (Trojovská, Dehghani & Trojovský, 2022). It also adopts the adaptive search step mechanism of the zebra algorithm to automatically adjust the search scope, enabling a smooth transition from wide exploration to fine local search. Furthermore, the ICS algorithm introduces the zebra algorithm’s probabilistic selection mechanism to flexibly switch search strategies, increasing diversity and avoiding local optima. These innovations significantly enhance the ICS algorithm’s global search capability, local fine-tuning, convergence speed, and optimization efficiency. The complementarity of the ZOA and CS algorithms also improves the ICS algorithm’s adaptability to complex, multimodal, and high-dimensional problems. The proposed ICS algorithm is tailored for financial time series forecasting, particularly in predicting arbitrage spreads. It initializes the population using a tent chaotic mapping to boost global search (Li, Han & Guo, 2020). The Cuckoo and Zebra algorithms’ synergy enables precise tuning of LSTM hyperparameters, crucial for accurate financial forecasting. The following are the key contributions of this work:

1) We replaced the Lévy flights in the cuckoo algorithm with a “zebra” pattern search strategy that incorporates the zebra algorithm’s adaptive search step mechanism and probabilistic strategy selection. This enhances the algorithm’s global exploration, local exploitation, convergence speed, and optimization efficiency while reducing the risk of getting stuck in local optima.
2) We propose an innovative ICS-LSTM network model optimized using the ICS for hyperparameter tuning. This model enables adaptive training with diverse financial time series data, specifically enhancing the accuracy of arbitrage spread predictions.

The remainder of this article is divided into several sections: “Data Analysis” presents the data analysis, which primarily focuses on the effectiveness of real data. “Materials and Methods” introduces the ICS algorithm and the ICS-LSTM network. “Results and Discussion” presents the experimental description and results analysis of ICS-LSTM and other models for intercommodity spread prediction. “Conclusions” concludes this article.

## Data analysis

In this section, we provide a clear and concise description of the data. And, we also affirm its validity and applicability through correlation analysis and the Engle-Granger (EG) cointegration test (Ssekuma, 2011).

The data used in this study was sourced from the Shanghai Futures Exchange in China. The exchange provides a snapshot-based order feed using the CTP protocol, which aggregates changes over the last 500 ms. The corresponding rebar and hot-rolled coil contracts’ 500-ms tick data were used to calculate the spread data. The final output is 1-min K-line data. Furthermore, since each contract has a duration of one year, we combined the historical data for the January, May, and October contracts of each year based on turnover to obtain the continuous spread data of the main contract. Ultimately, we acquired the spread data from 21:01 on July 15, 2020, to 10:50 on March 23, 2023, comprising a total of 225,155 data points over 654 days.

Before initiating inter-commodity spread trading, it is crucial to confirm the existence of a long-term stable cointegration relationship among the selected futures contracts. For this reason, we conducted a cointegration analysis of the closing prices of rebar and HRC. First, we conducted unit root tests on the closing prices of the two contracts.

From Table 2 we know that the two logarithmic series contain a unit root, *i.e*., they are non-smooth series, meanwhile, the unit root test is carried out on their differences, and the first-order differences of the two pairs of series do not contain a unit heel, *i.e*., their first-order differences are smooth series. The following regression equation is constructed for the closing price data of hc and rb.

(1) $$rb = 0.876960672658*hc + 405.938874678 + {\mathop{\rm Re}}\; sid01.$$

**Table 2:** Unit root test.

Variety	Dickey-Fuller	*P*-value	Steady or not
hc	0.035680	0.6942	No
RB	−0.025008	0.6745	No
Δhc	−96.58328	0.0001	Yes
Δrb	−93.88820	0.0001	Yes

We then conducted a unit root test on the residuals of the model. From Table 3 the residual Resid01 does not contain a unit root and is a stable sequence. It can be concluded that the closing prices of hc and rb contracts are in line with the cointegration relationship.

**Table 3:** Residual series test.

Residual sequence	ADF test	*P*-value	Steady or not
Resid01	−7.235169	0.0000	Yes

## Materials and Methods

The ICS algorithm is designed to determine the optimal hyperparameters in this part. Further, we propose the ICS-LSTM network to adaptively tune the prediction model. The network eliminates the impact of individual subjectivity elements in traditional hyperparameter tuning and provides a reliable reference for constructing effective and efficient predictive models in the field of algorithmic trading.

### ICS algorithm

The CS algorithm is derived from modeling the breeding behavior of cuckoos. CS algorithms typically use a real-valued representation scheme, where the position of each cuckoo takes the form of a vector in a continuous search space. For a problem with D dimensions, the position of a cuckoo can be encoded as: $\mathop x\nolimits_i = \left[ {\mathop x\nolimits_{i1} ,\mathop x\nolimits_{i2} , \ldots \ldots ,\mathop x\nolimits_{iD} } \right]$, where $\mathop x\nolimits_{ij}$ denotes the $j$ th dimension of the first value of the $i$ cuckoo. The algorithm utilized a balanced combination of local randomized wandering and global exploratory randomized wandering, controlled by a switching parameter. The local randomized wandering can be expressed as:

(2) $$\matrix{ {{\bf x}_i^{t + 1} = {\bf x}_i^t + \alpha s \otimes H\left( {{p_a} - \epsilon } \right) \otimes \left( {{\bf x}_j^t - {\bf x}_k^t} \right)} \cr }$$

where ${\bf x}_j^t$ and ${\bf x}_k^t$ are two solutions chosen randomly by random permutations, *H* is the Heaviside function that turns a discontinuous signal into a continuous function, thus facilitating analysis and processing. $\epsilon$ is a random number drawn from a uniform distribution, *s* is the step size, and $\otimes$ is the dot product operator. By generating a random matrix and comparing it to the parameter *pa*, if the random number is greater than *pa*, the corresponding position will return True (indicating retention), otherwise, it returns False indicating abandonment. On the other hand, global exploratory random wandering is achieved by Lévy flights, which are random wandering in random directions with step sizes derived from Lévy distributions. These Lévy flights are performed by animals and insects and are characterized by a series of straight flights followed by an abrupt 90-degree turn. The specific formulas are shown in Eqs. (3) and (4), where *α* > 0 is the step size scaling factor which is related to the size of the problem of interest, in most cases we can use *α* = 1. $\textstyle{1 \over {{s^{1 + \lambda }}}}$ is a power operation on *s* used to scale the step size. This determines the direction of the step and the size of the base. $\textstyle{{\lambda { \Gamma }\left( \lambda \right)\sin \left( {{{\pi \lambda } \over 2}} \right)} \over \pi }$ determines the direction of the step and the size of the base. $\lambda$ is a shape parameter (also known as the stability index or order) of the Lévy distribution that controls the hopping behavior of the Lévy flight. This parameter affects the thickness of the tail of the Lévy distribution, *i.e*., how often large jump steps occur.

(3) $$\matrix{ {{\bf x}_i^{t + 1} = {\bf x}_i^t + \alpha L\left( {s,\lambda } \right)} \cr } (0 \; < \;  \lambda \le 2)$$

(4) $$\matrix{ {L\left( {s,\lambda } \right) = \displaystyle{{\lambda { \Gamma }\left( \lambda \right)\sin \left( {\displaystyle{{\pi \lambda } \over 2}} \right)} \over \pi }\displaystyle{1 \over {{s^{1 + \lambda }}}},\left( {0 \; < \;  s \; < \;  1} \right)} \cr }$$

The CS uses random generation to initialize the population, which often results in uneven initialization and fails to cover the entire population. To address these issues, this article proposes using tent chaotic mapping to initialize the population. Tent chaotic mapping introduces randomness, traversal, and sensitivity to initial values, which can accelerate the algorithm’s convergence speed. This ensures that the initial solution is evenly distributed in the solution space. The comparison of the population distribution generated randomly and the population distribution initialized using tent mapping can be found in the Supplemental File. In both scenarios, the population size is set to 100, and after 30 independent experiments. The population distribution tends to be concentrated in one place for populations initialized using random search compared to populations initialized using tent chaotic mapping. Initializing the population using tent mapping leads to a more uniform population distribution. The tent map is a function with a parameter *µ* and is a segmented linear mapping. It is defined as shown in Eq. (5):

(5) $$\matrix{ {{x_{n + 1}} = \; {f_\mu }\left( {{x_n}} \right) = \; \left\{ {\matrix{ {\mu \mathop x\nolimits_n \quad\quad\quad\quad\quad {for \;x_n} \; < \;  \displaystyle{1 \over 2}} \cr {\mu \left( {1 - \mathop x\nolimits_n } \right){\rm }\quad\quad\quad{for\;x_n} \ge \displaystyle{1 \over 2}} \cr } } \right.} \cr }.$$

In the original CS algorithm, the Lévy flight mechanism is used to explore the solution space. As can be seen from Eqs. (3) and (4), the step size of the Lévy flight shows a pattern of frequent short distances and occasional long distances, and its search process allows individuals to easily span a wide range of search regions, resulting in a weak local search capability of the algorithm. When the algorithm needs to search for a local optimal solution, the randomness of the Lévy flight reduces the search efficiency, resulting in the algorithm needing more iterations to converge when approaching the optimal solution.

To solve the shortcomings of the original CS algorithm in terms of local search capability, we introduce ZOA to replace the Lévy flight mechanism while retaining the local search capability of the CS algorithm. ZOA is chosen because it has the following advantages: (1) balanced search strategy, which can strike a better balance between global exploration and local exploitation; (2) adaptive, which can automatically adjust the search strategy according to the current situation; and (3) utilizes group intelligence to guide the search, which can explore the complex solution space more effectively. The specific alternatives are: in the global search phase, the position update mechanism of ZOA is used instead of Lévy flight, so that individuals can move to the global and local optimal solutions more effectively; in the local search phase, the local search mechanism of CS algorithm is retained, which not only retains the advantages of CS algorithm, but also overcomes its deficiencies in local search, and improves the overall performance of the algorithm and the convergence speed.

ZOA also uses a real-valued representation scheme, where the position of each zebra is represented as a vector in a continuous search space: $\mathop x\nolimits_i = \left[ {\mathop x\nolimits_{i1} ,\mathop x\nolimits_{i2} , \ldots \ldots ,\mathop x\nolimits_{iD} } \right]$. Where $\mathop x\nolimits_{ij}$ denotes the *j*th dimension of the first value of the $i$ zebra. The zebra search involves two main phases. In the first phase, population members simulate zebra behavior while foraging for food. The most outstanding member of the ZOA acts as the pioneer zebra, guiding others to their positions in the search space. Equations (6) and (7) mathematically model the updating of pioneer zebras’ positions during the foraging phase. Where “*r”* is a random number between 0 and 1, and “*I”* is a random value from the set. The second phase models zebra defense strategies against predators. Zebras evade lion attacks using zigzag and random lateral maneuvers. Against smaller predators like hyenas and dogs, zebras adopt a more aggressive strategy, rallying together to confuse and intimidate their attackers. The ZOA considers two scenarios with equal probability: in the first, a zebra hides near its location from a lion (modeled by *S1* in Eq. (8)), The search step size of ZOA is adaptively adjusted as the number of iterations increases this allows the algorithm to explore a large area in the early stages and focus more on localized fine-grained search in the later stages; in the second, zebras in the herd gather around an attacked zebra to form a defensive structure (modeled by *S2* in reference Eq. (8)). By introducing a probabilistic selection mechanism that switches between different search strategies, it helps to increase algorithmic diversity. In this context, “*t”* represents the number of iterations, “*T”* stands for the maximum number of iterations, and “*R*” is a constant set at 0.01. *P* is the switching probability of the two strategies. The value is a random number between 0 and 1, and *AZ* represents the state of the attacked zebra. When updating a zebra’s position, if the zebra has a better objective function value at the new position, that new position is accepted. Modeling this updating condition using Eq. (9).

(6) $$\matrix{ {x_{i,j}^{new,P1} = {x_{i,j}} + r \cdot \left( {P{Z_j} - I \cdot {x_{i,j}}} \right)} \cr } (0\; < \;p\; < \;1)$$

(7) $${\rm X}_{\rm i} = \left\{ \matrix{ {\rm X}_{\rm i}^{{\rm new,P1}}\hfill & {{\rm }F}_i^{new,P1} \; < \;  F_i \hfill \cr {{\rm X}_{\rm i}}\hfill & else\hfill}\right.$$

(8) $${x_{i,j}^{{\rm new,}P2} = \left\{ {\matrix{ {{S_1}:{x_{i,j}} + R \cdot \left( {2r - 1} \right) \cdot \left( {1 - \displaystyle{t \over T}} \right) \cdot {x_{i,j}}} \cr {{S_2}:{\rm }{x_{i,j}} + r \cdot \left( {A{Z_j} - I \cdot {x_{i,j}}} \right)} \hfill  \cr } \; \; \; \; } \right.} (0\; < \;r\; < \;1)$$

(9) $${X_i} = \left\{ {\matrix{ X_i^{new,P2}\hfill & F_i^{new,P2} \; < \;  F_i\hfill \cr X_i \hfill & else \hfill } } \right..$$

To improve the performance of the CS algorithm, in this study, not only did we use tent chaotic mapping to initialize the population, but we also used the idea of the ZOA algorithm in the position update process of the CS algorithm. Updating of the bird’s nest can be like the zebra in the ZOA algorithm, which always pursues the current optimum and the global optimum. This not only maintains the stochasticity required for searching but also reduces the blindness of searching and speeds up the convergence of the population to the optimal solution, and at the same time, the stochastic elimination mechanism of the CS algorithm makes the algorithm able to smoothly escape from the local optimum, thus significantly enhancing its overall performance. The pseudo-code for the algorithm is shown in Box 1. This pseudo-code describes the optimization process of ICS. First, the algorithm receives information about the optimization problem and sets the number of iterations T and the population size N. It initializes the positions of the zebras and evaluates these positions utilizing an objective function. In each iteration, the algorithm updates the locations in two parts: the first part uses ZOA, which simulates the zebra’s foraging behavior and defense strategy, For each nest, the algorithm uses a new location inspired by the ZOA to generate a new location, *i.e*., the location of the pioneer zebra, and then, the algorithm searches around the pioneer zebra, after which it introduces a probabilistic selection mechanism that balances between two search strategies (when Ps < 0.5) and (when Ps >= 0.5). When Ps < 0.5, a careful local search is performed in the neighborhood of the current solution through an adaptive step size and a random search, and when Ps >= 0.5, an extensive exploration is performed in the whole search space by introducing a random factor and a diverse search; the second part uses CS, which updates the cuckoo’s locations based on the probability of discovery pa (set to 0.25). After each iteration, the current best solution is saved, and the best solution found in all iterations is finally output. The overall time complexity of the ICS algorithm is: $O(T*({\rm n}*\dim + n*f))$, $T$ is the total number of iterations, $n$ is the number of cuckoos, $\dim$ is the positional dimension of each cuckoo, $f$ is the complexity of computing adaptation. In numerous models for hyperparameter optimization, the MSE or MAE of the validation set is typically employed as the fitness function of the model. In this study, we selected MSE as the fitness function.

**Box 1:** Algorithm pseudo-code.

Integrated Cuckoo Search Algorithm (ICS)
Start ICS
1. Input: The optimization problem information.
2. Set the number of iterations (T) and the number of populations (N).
3. Initialization of population position using Eq. (5) and evaluation of the
objective function.
4. **Part1: Update position using ZOA**
5. For t = 1: T
6. Update Pioneer Zebra (PZ).
7. For i = 1: N
8. **Phase 1: Zebra foraging behavior**
9. Calculate the new status of the ith zebra using Eq. (6).
10. Update the ith zebra using Eq. (7).
11. **Phase 2: Defense strategies against predators**
12. If Ps < 0.5, Ps = rand
13. Strategy 1: against lion
14. Calculate the new status of the ith zebra using mode S1 in Eq. (8).
15. else
16. Strategy 2: against other predators
17. Calculate the new status of the ith zebra using mode S2 in Eq. (8).
18. end if
19. Update the ith zebra using Eq. (9).
20. **Part2: Update position using CS**
21. Set the probability of discovery pa to 0.25
22. Update the position of cuckoo birds using Eq. (2)
23. end for i = 1: N
24. Save the best candidate solution so far.
25. end for t = 1: T
26. Output: The best solution obtained by ICS for the given optimization problem.
End ICS.

### ICS-LSTM

#### Structure of ICS-LSTM

**Figure 1:**
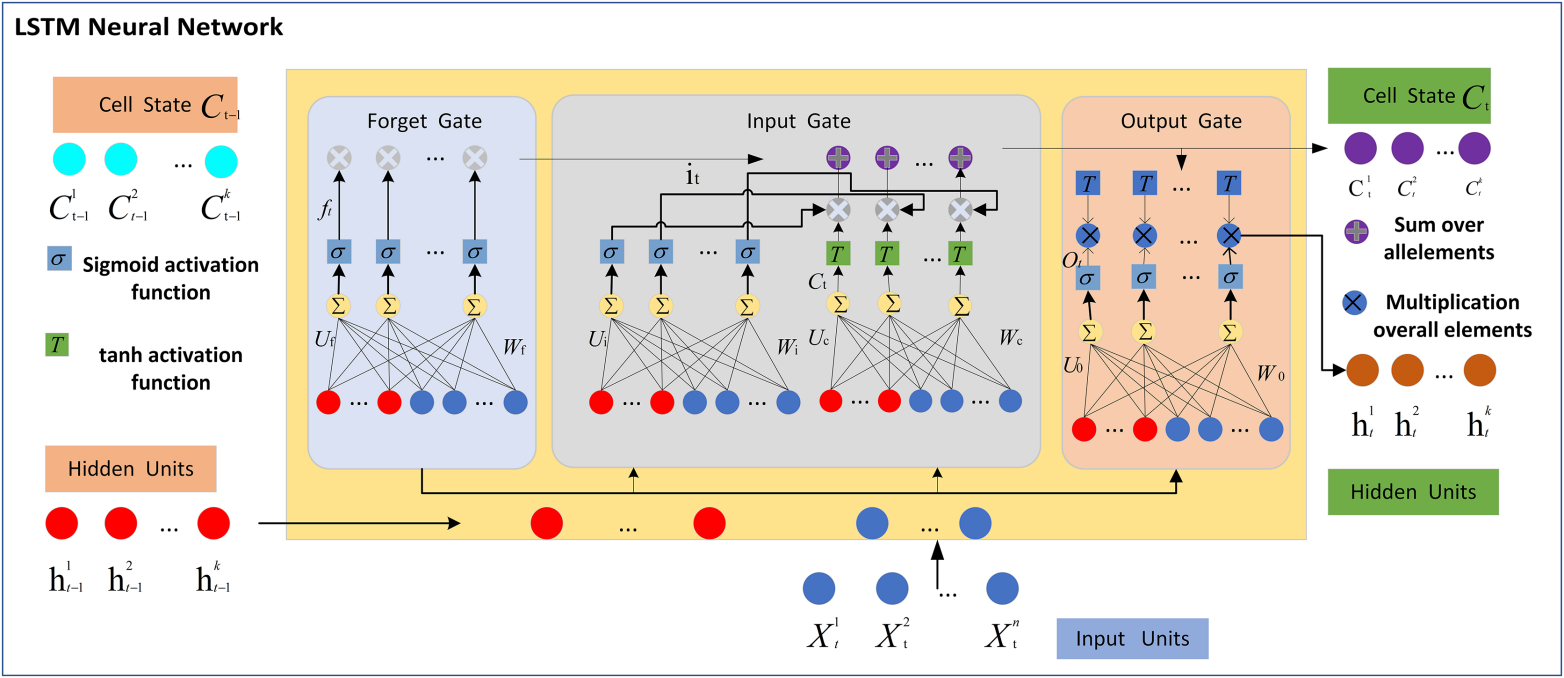
LSTM neural network.

The LSTM network is made up of the memory cell and three gates, namely the input gate, forget gate, and output gate. The three gates control the flow of information within the memory cell by determining what information to discard, store, and transmit to the next state. Figure 1 displays the typical structure of an LSTM network with a single hidden layer. The core part of the LSTM unit contained in the hidden layer(s) is the cell state. Let $\mathop X\nolimits_t = \left[ {x_t^1,x_t^2,x_t^3, \ldots \ldots x_t^N} \right]$ be the N number of inputs, $\mathop H\nolimits_t = \left[ {h_t^1,h_t^2,h_t^3, \ldots \ldots {\rm h}_t^K} \right]$ be the K hidden units or hidden states, and $\mathop C\nolimits_t = \left[ {c_t^1,c_t^2,c_t^3, \ldots \ldots c_t^K} \right]$ be the cell state of the LSTM network at time t and ${f_t}$, ${I_t}$ and ${O_t}$ represent the forget gate, input gate, and output gate, respectively. At each timestamp t, the input ${X_t}$ along with the previously hidden state $h_{t - 1}^1$ is presented to three gates to compute the next hidden state ${h_t}$ and to update the previous cell state ${C_{t - 1}}$ to compute the new cell state ${C_t}$. Where ${W_f}$, ${W_i}$, ${W_c},{W_o}$ are the weights matrices corresponding to the input ${X_t}$ and ${U_f}$, ${U_i}$, ${U_c}$, ${U_c}$ are recurrent weights matrices associated with previously hidden state ${h_{t - 1}}$ and ${b_f}$, ${b_i}$, ${b_c}$ and ${b_o}$ are the bias vectors for forget gate, input gate, candidate solution, and output gate, respectively. $\sigma \left( x \right)\;$ is a log-sigmoid activation function and ${\rm tanh}\left( x \right)$ is the hyperbolic tangent activation function.

**Figure 2:**
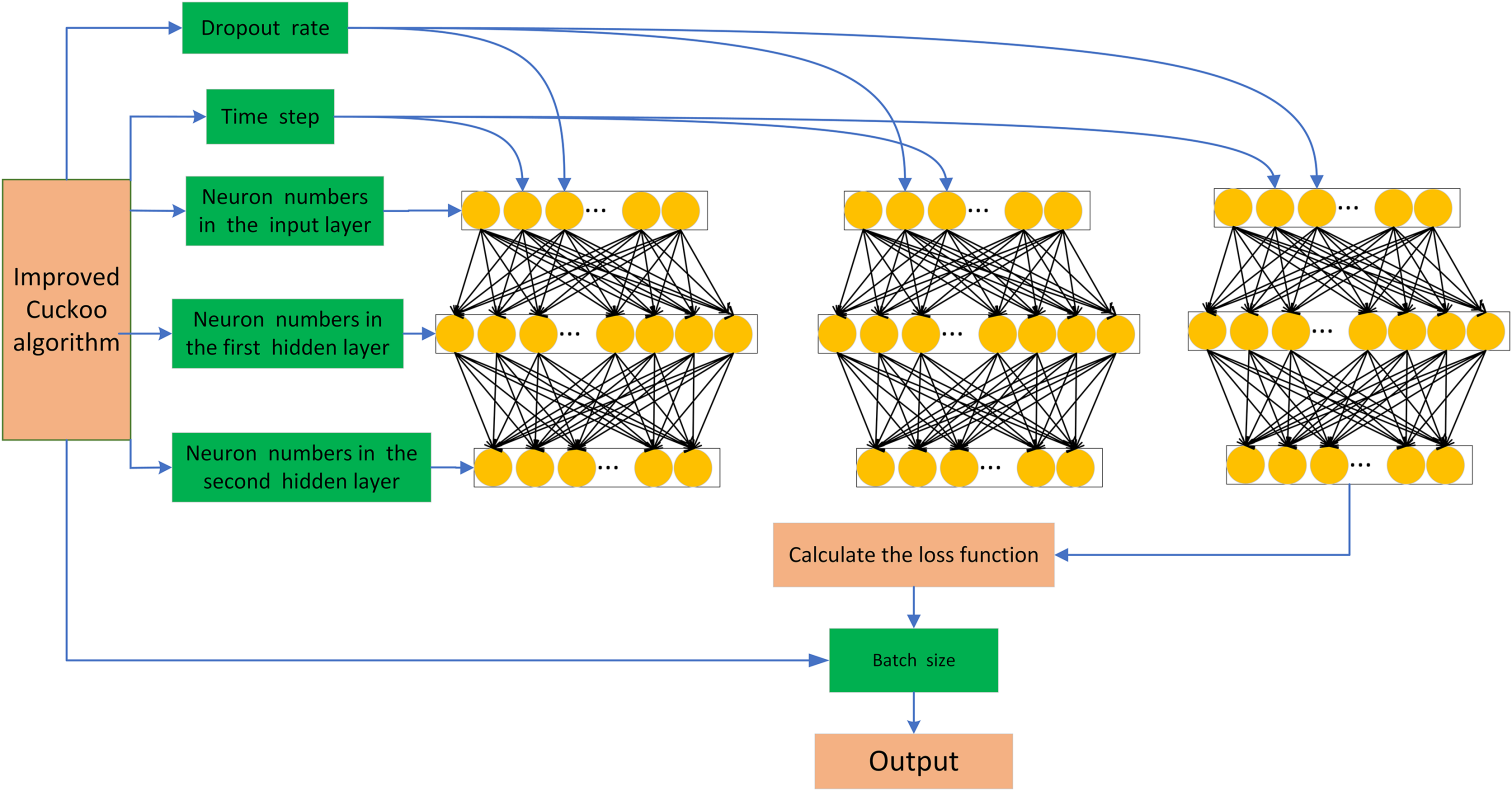
ICS-LSTM model architecture.

The ICS algorithm proposed in this article offers strong global search capability, high search accuracy, and fast convergence speed. In this article, we focus on optimizing the hyperparameters of the network model to minimize the influence of human factors on the network model and enhance the predictive ability of the model. Therefore, we consider the time step, discard rate, and the number of neurons in one input layer and two hidden layers of LSTM as the target optimization parameters. Finally, we establish the ICS-LSTM model with the structure shown in Fig. 2 for futures spread prediction.

#### Steps of the ICS

The specific process of ICS to optimize the parameters of the LSTM network is divided into the following five steps:

Step 1: Preprocessing. The data preprocessing consists of multiple sections: determining input features and normalization processing. After completing these tasks, we divide the training set and test set proportionally.

Step 2: Population and parameter settings. Set the number of populations, search scope, number of algorithm iterations, and generate the search space according to the number of cuckoos and the target parameters (discard rate, number of neurons, and time step). Initialize the population using tent mapping.

Step 3. Calculate the fitness value. We use mean squared error (MSE) as the fitness function to identify the best individuals based on their fitness values.

Step 4. Location update. Update the position of the population according to Eqs. (6)–(8) and (2). Then, compare the current global optimal solutions and adjust the optimal fitness value.

Step 5. Determine the loop situation. If the optimal fitness value is stabilized or the iteration is completed, output the best value; else, return to Step 3.

More details are available in the Supplemental File.

## Results and discussion

### Test function

We selected the CS and the ZOA, along with the enhanced Cuckoo Algorithm proposed in this article, for testing in the CEC 2019 test function. In CEC 2019, the F1-F3 functions have varying dimension values and ranges, while the F4-F10 functions are 10-dimensional minimization problems. All these optimization benchmarks aim to minimize the target fitness value. Since the search range of CEC-07 is consistently negative during the testing of the three algorithms, which does not align with the requirements of futures spread prediction models, all CEC 2019 function sets except CEC-07 have been chosen. The function introductions of the selected function sets can be found in the Supplemental File. The mean and standard deviation of the three algorithms after 30 independent runs under the nine test functions are shown. From Table 4 it can be seen that the enhanced cuckoo algorithm proposed in this article consistently outperforms the other two algorithms in terms of mean value. The standard deviation of the cuckoo algorithm in the functions F4, F5, F6, and F8 is the smallest among the three algorithms. The ZOA shows the best performance in the functions F2 and F10, while the improved cuckoo algorithm proposed in this article excels in the functions F1, F3, and F9, performing only slightly worse than the other two optimal algorithms in the remaining test functions.

**Table 4:** Statistical results of ICS compared to other algorithms on classic benchmarks.

Functions	Indicator	Algorithms		
		CS	ZOA	ICS
F1	Mean	14,222.621	1.0	1.0
	Std Dev	7,098.222	0.0	0.0
F2	Mean	203.817	5.0	4.913
	Std Dev	51.121	0.0	0.224
F3	Mean	7.038	6.961	3.069
	Std Dev	0.685	0.686	0.407
F4	Mean	23.166	14,023.811	21.115
	Std Dev	3.431	5,702.329	3.490
F5	Mean	1.125	4.754	1.115
	Std Dev	0.023	0.607	0.027
F6	Mean	8.880	10.242	8.527
	Std Dev	0.472	0.707	0.781
F8	Mean	1.000	1.457	1.00
	Std Dev	7.152e−15	0.125	3.913
F9	Mean	1.278	558.604	1.262
	Std Dev	0.044	166.737	0.043
F10	Mean	21.052	21.413	14.612
	Std Dev	1.030	0.106	6.716

### Data processing

The 1-min period K-line fitted spread data of rebar and HRC futures on the Shanghai Futures Exchange from December 34, 2020, to March 15, 2023, was used to evaluate the proposed prediction model. Each period’s price spread data includes eight features: opening price spread, highest price spread, lowest price spread, closing price spread, MACD, DEA, DIF, and price spread fluctuation. The closing price spread is the target for prediction.

Data preprocessing is required before training, and the data preprocessing steps include the following: firstly, data cleaning is performed to remove missing values and error records, and forward or backward padding is used to ensure the continuity of the time series for missing data during non-trading hours; next, min-max normalization is performed on all the features to ensure that the numerical scales of the different features are consistent, to avoid affecting the learning effect of the model; then, the data is divided into a training set (80%), a validation set (10%), and a test set (20%), which are used for model training, hyper-parameter tuning, and final evaluation, respectively; and lastly, technical indicators such as MACD, DEA, and DIF are calculated using the historical price data, which are used as the input features to help the model capture the market trend and the time series pattern. These preprocessing steps ensure the quality and consistency of the data and provide a reliable basis for model training and comparison.

### Metrics

To demonstrate the predictive effectiveness of each model, four metrics were used to measure the performance of each model: MSE, MAPE, MAE, and coefficient of determination ( ${R^2}$). This is calculated by Eqs. (10) to (13).

(10) $$MSE = \displaystyle{1 \over n}\sum\limits_{i = 1}^n {\mathop {\left( {{{\hat{\rm y}}_{\rm i}} - \mathop y\nolimits_i } \right)}\nolimits^2 }$$

(11) $$\matrix{ {MAE = \displaystyle{1 \over {\rm n}}\mathop \sum \limits_{{\rm i} = 1}^{\rm n} \left| {{{\hat{\rm y}}_{\rm i}} - {{\rm y}_{\rm i}}} \right|} \cr }$$

(12) $$\matrix{ {MAPE = \displaystyle{{100{\rm \% }} \over n}\mathop \sum \limits_{i = 1}^n \left| {\displaystyle{{{{\hat{\rm y}}_{\rm i}} - {y_i}} \over {{y_i}}}} \right|} \cr }$$

(13) $$\matrix{ {\mathop R\nolimits^2 = 1 - \displaystyle{{\mathop \sum \nolimits_i {{\left( {{{\hat{\rm y}}_{\rm i}} - {y_i}} \right)}^2}} \over {\mathop \sum \nolimits_i {{\left( {\overline {{y_i}} - {y_i}} \right)}^2}}}} \cr }$$

where ${y_i}$ is the predicted output value of the ith observation of the model, ${\hat{\rm y}_{\rm i}}$ is the expected value, and n denotes the number of samples. If the value of MAE, MSE, and MAPE is smaller, the smaller the deviation between the predicted value and the original value. Also the closer the coefficient of determination $\mathop R\nolimits^2$ is to 1, the better the model fits. In addition to the aforementioned indicators, one traditional assessment indicator has been employed, as defined in Eq. (14).

(14) $$SA = \displaystyle{{\sum\limits_{t = 1}^n {\left( {{{\hat y}_t} - \bar{\hat y}} \right)\left( {{y_t} - \bar{ y}} \right)} } \over {\sqrt {\sum\limits_{t = 1}^n {{{\left( {{{\hat y}_t} - \bar{\hat y}} \right)}^2}} } \sqrt {\sum\limits_{t = 1}^n {{{\left( {{y_t} - \bar{ y}} \right)}^2}} } }}$$

where ${z_t} = \left\{ \matrix{\matrix{ {1,} & {if} & {\left( {{y_{t + 1}} - {y_t}} \right)\left( {{{\hat y}_{t + 1}} - {y_t}} \right)} \cr } \; > \;  0 \hfill \cr \matrix{ {0,} & {if} & {\left( {{y_{t + 1}} - {y_t}} \right)\left( {{{\hat y}_{t + 1}} - {y_t}} \right) \; < \;  0} \cr } \hfill} \right.$; symbol accuracy focuses on the correctness of the trend direction, which is more important in arbitrage scenarios than precise numerical predictions.

### Model implementation

To enhance the efficiency of updating network weights, the Adam optimizer was selected. The Adam optimizer adaptively adjusts the learning rate of each parameter based on historical gradient information, improving the efficiency and stability of the training process. The number of cuckoo populations was set to 10, and the discovery probability for the search was set to 0.25. Five hyperparameters of the LSTM model require optimization: dropout rate, input layer neurons, hidden layer neurons, and time step.

We established a reasonable search range for the parameters to prevent potential issues during the search process. We analyzed research articles (Chen, Zhou & Dai, 2015; Ding & Qin, 2020; Lu et al., 2021) on LSTM models for forecasting the Chinese futures arbitrage market and set the search range of parameters as presented in Table 5. The search range of the time step was set to 3 to 20, the discard rate to 0.002 to 0.99, and the number of neurons for all three layers to 1 to 200. After optimizing the improved cuckoo algorithm, the final optimal parameters are three for the time step, 0.468 for the discard rate, and 109 neurons for the input layer. The number of neurons in the first hidden layer is 107, and the second hidden layer is 98. More on the optimization process can be found in the Supplemental File.

**Table 5:** Parameter search range.

Parameter	Search range	Optimal value
Time step	[1, 20]	3
Dropout rate	[0.002, 0.99]	0.468
Neuron numbers in the input layer	[1, 200]	109
Neuron numbers in the first hidden layer	[1, 200]	107
Neuron numbers in the second hidden layer	[1, 200]	98

### Experimental results and analysis

**Figure 3:**
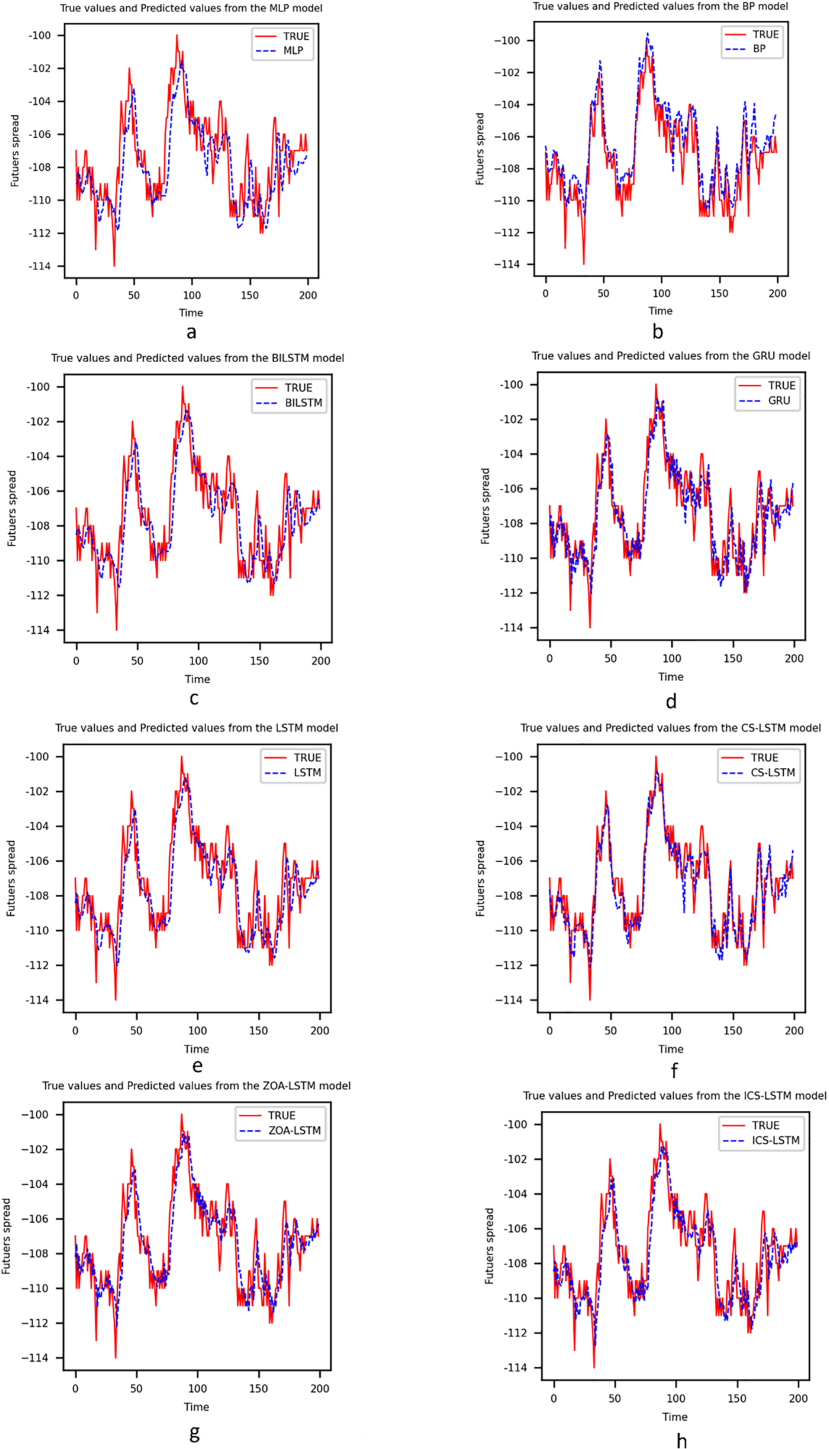
(A–H) Comparison between prediction results of each model.

This section analyzes the capability of ICS-LSTM for predicting spread trends. Models such as multilayer perceptron (MLP), back propagation (BP), LSTM, BiLSTM, and gated recurrent unit (GRU) are widely used in the field of futures arbitrage prediction (Nosratabadi et al., 2021; Zhang et al., 2022; DiPietro & Hager, 2020; Siami-Namini, Tavakoli & Namin, 2019; Farah et al., 2022). MLP and BP are classic neural network models widely used in early time series prediction tasks. However, these models have some limitations. MLP and BP models have a large number of parameters and are prone to overfitting, and they also struggle to capture the long-term dependencies in time series data. In contrast, the ICS-LSTM method introduces the ICS optimization algorithm, which can effectively avoid local optima and improve the model’s search capability and generalization ability. Therefore, the ICS-LSTM method is more suitable for handling complex time series data than MLP and BP methods. LSTM, BiLSTM, and GRU are advanced time series prediction models that can capture the long-term dependencies in the data. However, the optimization process of these models is prone to getting trapped in local optima, leading to poor model performance. The ICS-LSTM method, by introducing the ICS optimization algorithm, can automatically adjust the model’s hyperparameters and improve the model’s search capability and generalization ability. CS-LSTM and ZOA-LSTM are methods that use CS and ZOA algorithms to optimize the LSTM model. These methods can effectively improve the performance of the LSTM model. However, both CS-LSTM and ZOA-LSTM methods have some limitations, and it is difficult to achieve accurate prediction of arbitrage price difference. In contrast, the ICS-LSTM method can automatically adjust hyperparameters and effectively avoid local optima, outperforming other time series forecasting models in the task of arbitrage spread prediction. The main experimental results are shown in Table 6 and Fig. 3.

**Table 6:** Evaluation results of models.

Model	MSE	MAPE	MAE	R2	SA
ICS-LSTM	3.326	0.011	1.261	0.996	0.4714
GRU	4.218	0.023	1.574	0.994	0.4013
LSTM	14.984	0.017	3.410	0.980	0.3747
CS-LSTM	3.705	0.012	1.391	0.995	0.4469
ZOA-LSTM	5.721	0.017	1.791	0.992	0.4526
BP	7.760	0.022	2.483	0.989	0.4532
MLP	8.620	0.016	2.180	0.989	0.3566
BILSTM	8.922	0.013	2.397	0.988	0.3832

To be specific, although MLP and BP networks can roughly predict future trends, they are unable to cope with drastic price fluctuations, and there are obvious differences between the prediction curve and the actual curve, especially at the inflection points. The MLP is a basic feed-forward artificial neural network, and it has an MSE of 8.620, a MAPE of 0.016, an MAE of 2.180, and an R2 of 0.989, from the prediction curve and evaluation indexes, it is obvious that the prediction of MLP is not accurate enough; BP is a classical artificial neural network model, and its MSE is 7.760, MAPE is 0.022, MAE is 2.483, and R2 is 0.980, which can be seen that BP’s MSE is better than MLP, but other indexes are not as good as MLP, which indicates that the overall prediction indexes of BP are slightly better than those of MLP. Although the overall prediction index of the BP network is slightly better than that of MLP, the prediction error of the BP network is rather larger in some key data points. The above results show that there are obvious deviations between MLP and BP networks in predicting future trends, especially when dealing with inflection points and drastic fluctuations.

Compared to traditional models like MLP and BP, LSTM excels at capturing time dependencies and reducing prediction errors, thanks to its gating mechanism and memory cells. However, its performance depends heavily on hyperparameter selection, with metrics showing room for improvement: MSE is 14.984, MAPE is 0.017, MAE is 3.410, and R2 is 0.980. In contrast, the error indicators of GRU are 4.218 for MSE, 0.023 for MAPE, 1.574 for MAE, 4.218 for MSE, and 0.994 for R2. It can be seen that the overall prediction indicators of GRU are better than those of LSTM, but the indicator of MAPE is slightly higher, which indicates that the selection of hyperparameters also has a certain effect on the GRU model. However, the combined prediction curves and indicators show that GRU is better than LSTM in terms of prediction accuracy and stability. BiLSTM enhances LSTM by taking into account both forward and backward information in the input sequence to better understand the context. The metrics are as follows: the MSE is 8.922, the MAPE is 0.013, the MAE is 2.397, and the R2 is 0.988. It can be seen that BiLSTM outperforms the LSTM, but its overall error MSE is still somewhat high. After applying the swarm intelligence optimization algorithm to optimize the model parameters, we found that the optimized model has better generalization and fitting ability. The values of MSE, MAPE, MAE, and R2 are 3.705, 0.012, 1.391, and 0.995 for the CS-LSTM. For the ZOA-LSTM, the values are 5.721, 0.017, 1.791, and 0.995 respectively. From the results, it can be seen that both algorithms optimize the LSTM model with significant improvement compared to the baseline LSTM, and the prediction results are more in line with the real curves. Among them, the results of CS-LSTM are more optimized for ZOA-LSTM, indicating that the global search capability of the CS algorithm performs better in the optimization search process.

The MSE, MAPE, and MAE values of the ICS-LSTM model are 3.326, 0.011, and 1.261 respectively. Compared with MLP, BP, LSTM, BiLSTM, GRU, CS-LSTM, and ZOA-LSTM models, the MSE of the ICS-LSTM model is reduced by 61.4%, 57.1%, 77.8%, 62.7%, 21.1%, 10.2%, and 41.8%, respectively. Additionally, the MAPE is reduced by 31.2%, 50%, 35.2%, 15.3%, 52.1%, 8.3%, and 35.2%, respectively, compared to the other seven models. And the MAE is reduced by 42.1%, 49.2%, 63.0%, 47.3%, 19.9%, 9.3%, and 29.6%, respectively. The results for all three indicators indicate that the error between the predicted and actual values of ICS-LSTM is smaller compared to other models, resulting in higher prediction accuracy than other models. In the results of the model evaluation presented in Table 6, the R2 value of ICS-LSTM is the closest to one compared to all other models, suggesting that the model has a stronger fitting ability. All the experimental results show that the predictive ability and prediction effectiveness of the ICS-LSTM model are superior to other methods.

To more rigorously evaluate the performance of the ICS-LSTM model in predicting future arbitrage trends, we introduced the metric of symbol accuracy (SA). The model’s effectiveness in forecasting future price trends is determined by calculating the proportion of predicted values that are symbolically aligned with actual values. Experimental results indicate that the ICS-LSTM model achieves a symbol accuracy of 47.1%, which is notably higher than that of the unoptimized LSTM model (37.4%) and exceeds the performance of other comparative models. This suggests that the ICS-LSTM model effectively captures both upward and downward market movements. By accurately predicting price trends, investors can better conduct arbitrage trades and maximize returns. At the same time, the high symbol accuracy of the ICS-LSTM model can also help investors avoid losses due to price fluctuations. Therefore, the application of the ICS-LSTM model in the futures arbitrage market has great potential and value.

Analysis shows that LSTM architectures capture long-term dependencies in time series data due to their memory units and gating mechanisms. This capability allows LSTM to perform well when dealing with data exhibiting long-term trends or repetitive patterns. However, the model’s performance is highly contingent on the selection of hyperparameters, such as the number of neurons, time steps, and dropout rates. The ICS method enhances the traditional cuckoo algorithm by introducing a more flexible search mechanism, thus avoiding local optima in the complex optimization space, which is critical for improving the prediction accuracy of LSTM models. By leveraging the LSTM’s robust handling of time-series data and the efficiency of the improved cuckoo algorithm in hyperparameter optimization, the ICS-LSTM model demonstrates a significant enhancement in futures arbitrage prediction accuracy.

## Conclusions

This study develops an LSTM-based futures spread prediction model, named ICS-LSTM. The model optimizes the parameters of the LSTM network by integrating the cuckoo algorithm, which enhances the generalization ability and prediction effect of the model. Rebar and HRC 1-min K-line spread data from the Shanghai Futures Exchange are used for training and testing. Comparative experiments with LSTM and other methods verify the ICS-LSTM model’s high prediction accuracy for forecasting future spread trends.

However, there are still some limitations. Firstly, due to the limitation of hardware equipment, only a single set of future spread data was selected for experiments. Secondly, the current model only utilizes the LSTM network structure, without exploring integration with other advanced machine learning or deep learning algorithms like CNN or GNN, which could potentially improve prediction accuracy and robustness.

To address these limitations, future research can improve in several ways. First, more futures spread data should be used to comprehensively verify the ICS-LSTM model’s generality and improve its performance. Second, combining ICS-LSTM with advanced algorithms like CNN or GNN can be explored to enhance prediction accuracy and robustness. Additionally, applying the ICS-LSTM model to real-time financial market data prediction and trading strategy formulation can evaluate its performance in high-frequency trading and real-time decision-making, optimizing its real-market application.

## Supplemental Information

10.7717/peerj-cs.2552/supp-1 Supplemental Information 1 CEC19 Testing.

10.7717/peerj-cs.2552/supp-2 Supplemental Information 2 CEC 2019 test suite.

10.7717/peerj-cs.2552/supp-3 Supplemental Information 3 Flowchart of the ICS-LSTM.

10.7717/peerj-cs.2552/supp-4 Supplemental Information 4 Code for Improved Cuckoo Algorithm Optimised LSTMs.

10.7717/peerj-cs.2552/supp-5 Supplemental Information 5 Optimization process for each parameter.

10.7717/peerj-cs.2552/supp-6 Supplemental Information 6 Population initialization methods.

10.7717/peerj-cs.2552/supp-7 Supplemental Information 7 Data: optimization process for each parameter.

10.7717/peerj-cs.2552/supp-8 Supplemental Information 8 Source and Shanghai Futures Exchange spread data for rebar and HRC.

## Additional Information and Declarations

### Data Availability

The following information was supplied regarding data availability: The original code and data are available in the Supplemental Files.
