# Supplementary material for: Improving long short-term memory (LSTM) networks for arbitrage spread forecasting: integrating cuckoo and zebra algorithms in chaotic mapping space for enhanced accuracy
Source: PeerJ Comput Sci. 2024 Dec 12;10:e2552. doi: 10.7717/peerj-cs.2552 (PMC11784865; doi:10.7717/peerj-cs.2552)
Supplement: Supplemental Information 2 [file peerj-cs-10-2552-s002.docx]

**Table CEC 2019 test suite.**

| Func. | Descriptions | f_min | Search Range | Dim |
| --- | --- | --- | --- | --- |
| CEC-01 | Storn’s Chebyshev Polynomial Fitting Problem | 1 | [8192, 8192] | 9 |
| CEC-02 | Inverse Hilbert Matrix Problem | 1 | [16384, 16384] | 16 |
| CEC-03 | Lennard-Jones Minimum EnergyCluster | 1 | [4, 4] | 18 |
| CEC-04 | Rastrigin’s Function | 1 | [-100, 100] | 10 |
| CEC-05 | Griewangk’s Function | 1 | [-100, 100] | 10 |
| CEC-06 | Weierstrass Function | 1 | [-100, 100] | 10 |
| CEC-08 | Expanded Schaffer’s Function | 1 | [-100, 100] | 10 |
| CEC-09 | Happy Function | 1 | [-100, 100] | 10 |
| CEC-10 | Ackley Function | 1 | [-100, 100] | 10 |
