# Supplementary figures and images for: Improving long short-term memory (LSTM) networks for arbitrage spread forecasting: integrating cuckoo and zebra algorithms in chaotic mapping space for enhanced accuracy
Source: PeerJ Comput Sci. 2024 Dec 12;10:e2552. doi: 10.7717/peerj-cs.2552 (PMC11784865; doi:10.7717/peerj-cs.2552)

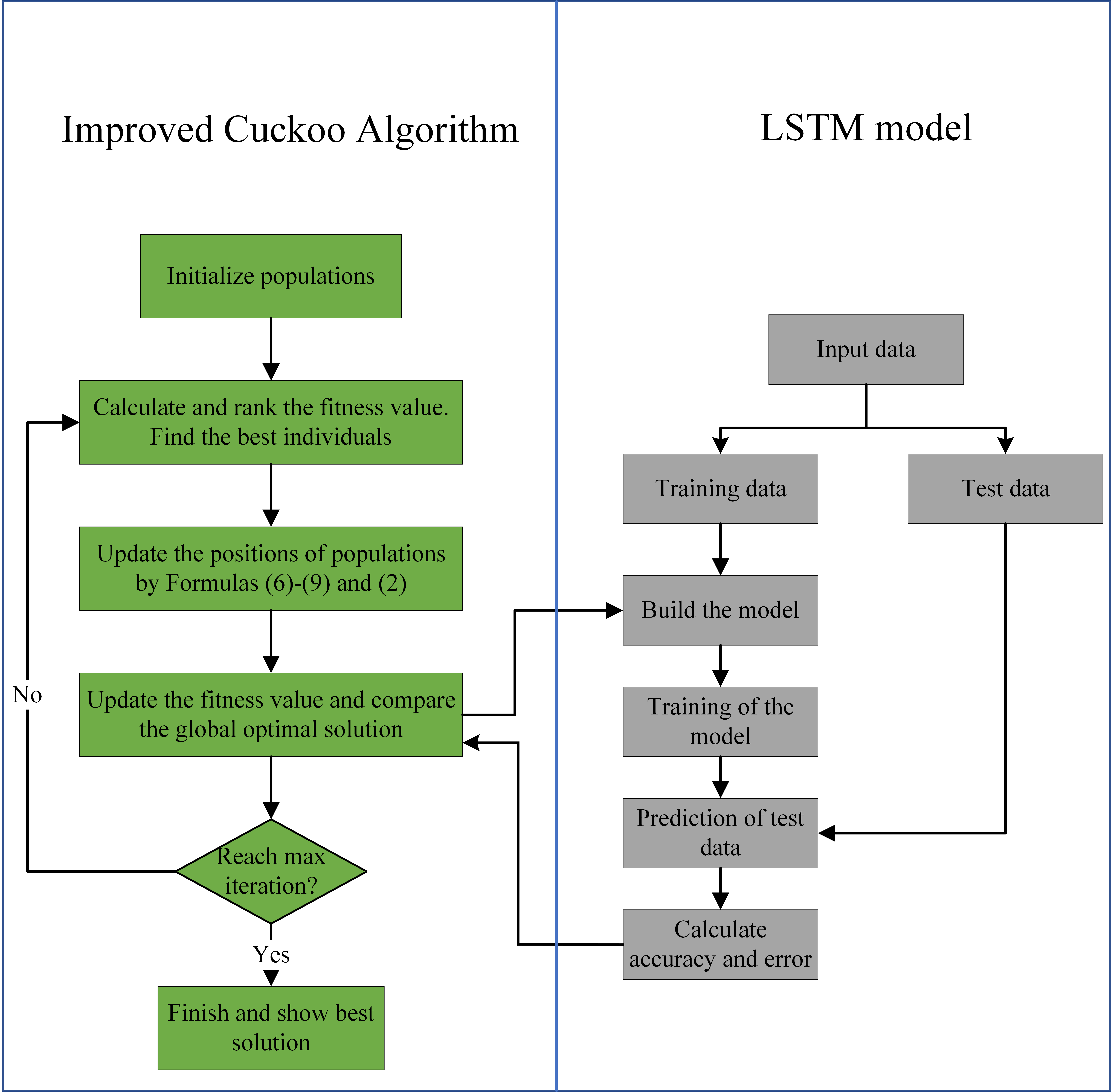

Supplement: Supplemental Information 3 [file peerj-cs-10-2552-s003.png]

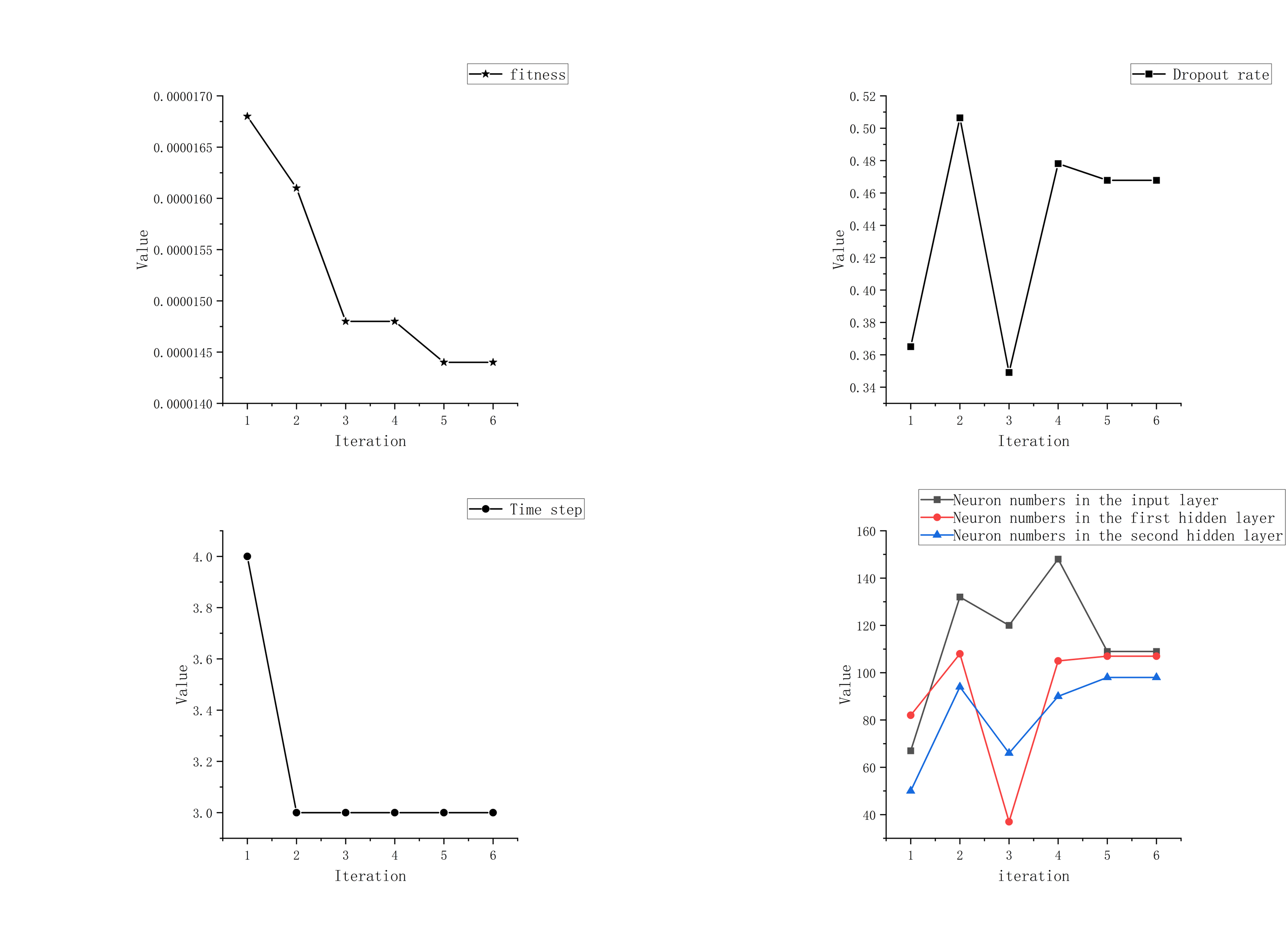

Supplement: Supplemental Information 5 [file peerj-cs-10-2552-s005.png]

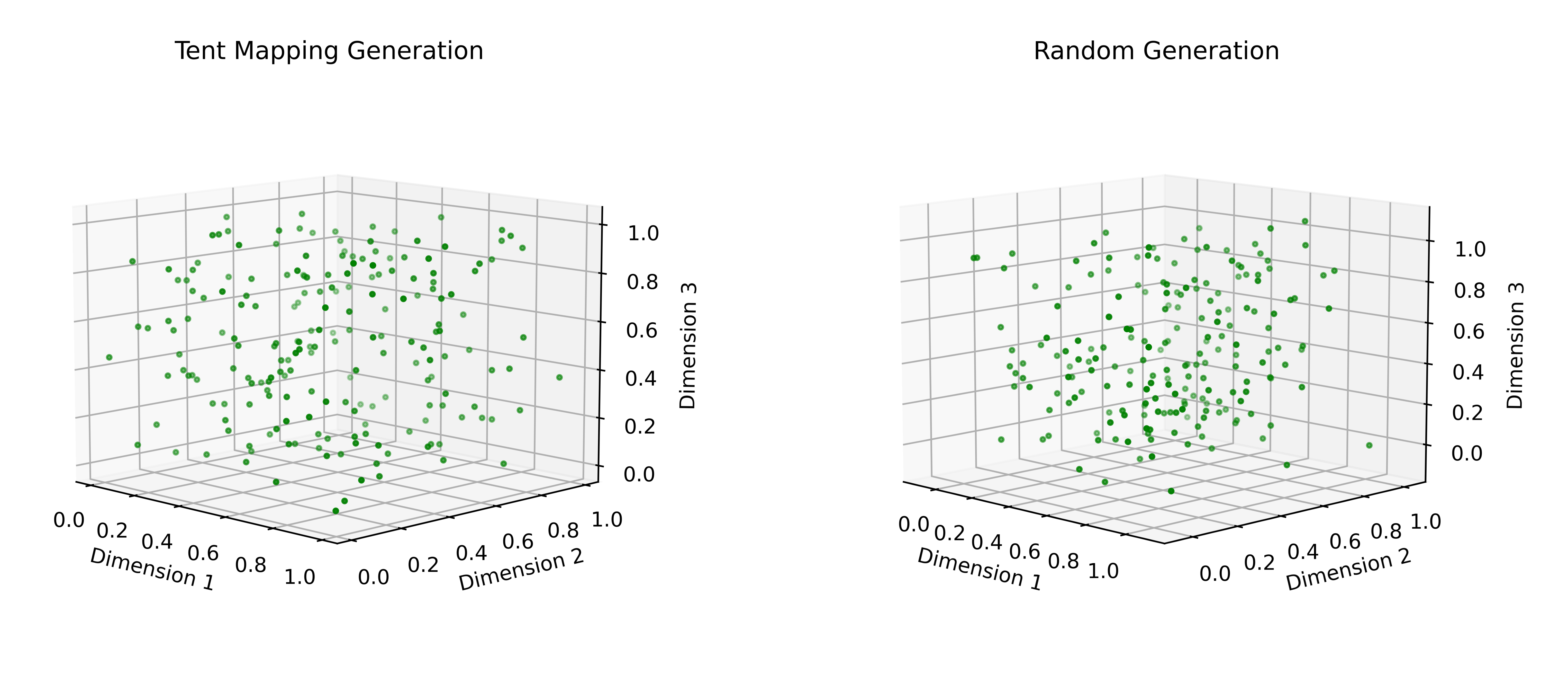

Supplement: Supplemental Information 6 [file peerj-cs-10-2552-s006.png]
