# Supplementary material for: Improving long short-term memory (LSTM) networks for arbitrage spread forecasting: integrating cuckoo and zebra algorithms in chaotic mapping space for enhanced accuracy
Source: PeerJ Comput Sci. 2024 Dec 12;10:e2552. doi: 10.7717/peerj-cs.2552 (PMC11784865; doi:10.7717/peerj-cs.2552)
Supplement: Supplemental Information 7 [file peerj-cs-10-2552-s007.docx]

**Table optimization process for each parameter.**

| Algorithm  iterations | Time  step | Dropout  rate | Neuron numbers in the  input layer | Neuron numbers in the first  hidden layer | Neuron numbers in the second hidden layer |
| --- | --- | --- | --- | --- | --- |
| 1 | 4 | 0.365 | 67 | 82 | 50 |
| 2 | 3 | 0.506 | 132 | 108 | 94 |
| 3 | 3 | 0.349 | 120 | 37 | 66 |
| 4 | 3 | 0.478 | 148 | 105 | 90 |
| 5 | 3 | 0.468 | 109 | 107 | 98 |
| 6 | 3 | 0.468 | 109 | 107 | 98 |
